# Structural, Optical, and Electrical Characterization of Biological and Bioactive Propolis Films

**DOI:** 10.1021/acsomega.2c05368

**Published:** 2022-11-16

**Authors:** Ferid Mezdari, Kamel Khirouni

**Affiliations:** Laboratory of Physics of Materials and Nanomaterials Applied at Environment, Faculty of Sciences, University of Gabes, 6072Gabes, Tunisia

## Abstract

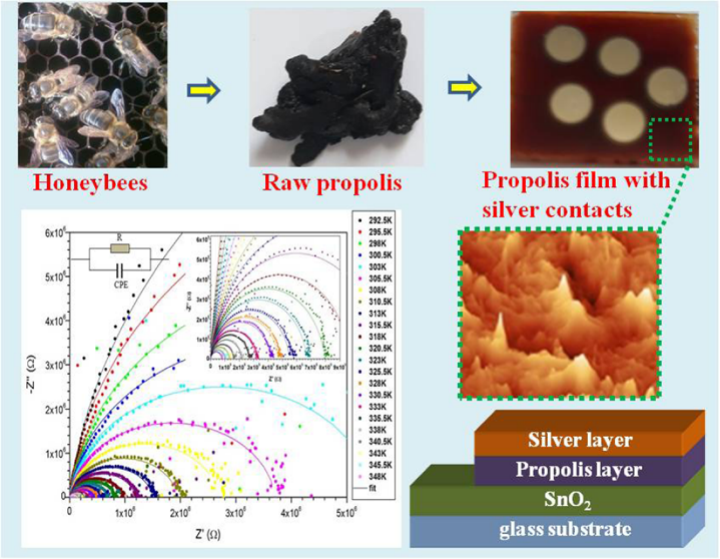

Natural substances are potential compounds for green electronic devices. So, scientists have to explore and optimize their properties to insert them as active layers in electronic heterostructures. In this study, microstructural, optical, and electrical properties of thin layers of the propolis are investigated. Propolis is a biological organic bioactive material produced by honeybees. A stable, bioactive, green, and low-cost thin layer of this biocompatible material was deposited on different substrates using a propolis alcohol solution. The morphological studies show that the propolis thin film is dense and well covers the substrate surfaces. Transmittance spectra show that propolis film cuts off blue and ultraviolet (UV) radiation, which are responsible for food oxidation, nutrient losses, flavor degradation, and discoloration. Therefore, to prevent food deterioration, a propolis film can be used in food packaging. For red and near-infrared radiation (∼600–2700 nm), a propolis film is transparent. Between near-infrared and mid-infrared radiation (∼2700–3200 nm), a propolis film reveals significant photosensitivity and so can be used as a photosensor. The propolis film reveals an energy gap of 2.88 eV at room temperature, which enables potential optoelectronic applications in the UV and blue ranges. The electrical study shows that the propolis layer has semiconductor behavior and can be a potential active layer in biocompatible temperature sensors. In addition to its medical, pharmaceutical, and food industry applications, in light of this study, propolis presents amazing optical and electrical properties and is a promising candidate for food packaging, optoelectronics, transparent electronics, and bioelectronics.

## 1 Introduction

Although electronic devices based on inorganic materials and classical semiconductors have reached a significant level of effectiveness and technological process, they still suffer from their high cost and polluting waste. To overcome these inconveniences, researchers are looking for new materials to develop low-cost green electronic devices. Organic materials, whether synthetic or natural, are of particular interest. In particular, biological materials have outstanding environmental and technical properties. Indeed, natural organic materials are eco-friendly, biodegradable, nontoxic, lightweight, mechanically flexible, and inexpensive, and they have potentially promising applications in logic and memory circuits, optoelectronics, photovoltaic devices,^1−9^ and in medical, pharmaceutical, and food industry.^10−12^ Furthermore, biological materials show efficient charge transport and a fast electrical and optical response, less than 5 ps.^13,14^ They present intense optical absorption and emission and mixed electronic and ionic conduction.^9^ Organic materials exhibit semiconductor behavior. Nevertheless, unlike inorganic semiconductors, no insulating oxide forms on their surface once exposed to the air. Heavy p- or n-doping leads to a remarkable change in their electronic structure.^9^ The development of organic light-emitting diodes is a potentially promising choice for the manufacturing of highly efficient and large light-source surfaces.^15−19^ The advances in organic light-emitting diodes^15−19^ have opened up interesting perspectives for the organic photovoltaic cell sector. Even if they have not achieved the efficiency of their inorganic competitors, they enjoy an easier and cheaper production process.^20−23^ Knowing this, providing sustainable and renewable energy sources is among the major issues facing humanity in the future.

Among these useful organic materials, we find the propolis complex (PC). PC is also called honeybee glue. It is a natural, organic, and bioactive material produced by honeybees. They use it to disinfect their honeycomb, mummify intruders, or seal cracks in their hive. PC is soft and sticky when warm but hard and brittle when cold and has a pleasant aromatic odor. This biologically active material is present in a large number of drugs because of its therapeutic properties. In fact, this natural complex material is made up of several biologically active molecules. The chemical composition of PC is very complex and depends on the botanical source and geographic origin. More than 300 active compounds have been found in raw propolis.^24,25^ PC is essentially composed of resins (50%), wax (30%), essential oils (10%), pollen (5%), and other organic compounds (5%).^25,26^ Among these organic compounds, there are phenolic and polyphenolic compounds, esters, flavonoids in their different forms (flavones, flavonoles, dihydroflavonoles, flavonones, chalcones), terpenes, β-steroids, aromatic aldehydes, sugars (fructose, glucose), and alcohols.^25,26^ PC has been used by worldwide people since immemorial times. In ancient Egypt, it was used for embalming the dead. A great deal of research on the biological activities of PC exists.^26−31^ Scientists have been interested in PC for its pharmaceutical properties and for many other virtues. PC possesses antibacterial, antiviral, antifungal, anti-inflammatory, anesthetic, antioxidant, antitumor (cytotoxic), immune-stimulating, wound-healing, and antiulcerogenic properties.^24−27,32,33^ The incorporation of PC in materials, such as polymers and natural rubbers, gives them a high potential for food and medical applications, protects them from the degradation of their functional parameters, and prevents a rapid decrease in their lifetimes.^34−38^ For instance, the mixture of natural rubber latex and PC makes it possible to produce highly flexible, translucent, nonadhesive, and biologically active membranes.^34,39^ These membranes can be used as an effective dressing for burns and wounds.^34,39^ Investigations, aimed at using PC as an eco-friendly corrosion inhibitor agent, have shown that PC acts as an anticorrosion agent on mild steel and copper in a high-salinity medium.^40,41^ Therefore, PC can be used as a protective coating of metals in chloride media or some aggressive environments in general. The synergistic effect of zinc oxide nanoparticles and ethanolic extract of propolis, deposited on bacterial cellulose, was investigated and gave rise to the formation of a biodegradable film with antimicrobial properties.^42^ However, there are few studies on the electrical and optical properties of this natural material. Drapak et al.^43^ created a heterojunction between indium monoselenide (InSe) and Ukrainian PC. The electrical properties of this heterojunction have been studied. Then, significant photosensitivity in the near-infrared range has been observed,^43^ and the p-type semiconductor behavior of the PC has been deduced.^43^ The optical properties of Ukrainian propolis films were investigated in the wavelength ranges λ = 350–1000 and 2750–3500 nm.^44−46^ The optical energy gap *E_g_* ≈ 3.07 eV was estimated from the absorption spectra and confirmed by a maximum of photoluminescence spectra at 2.86 eV.^44−46^ Thus, PC films possess the maximum quantum yield of photoluminescence in the blue region of the optical spectrum. Furthermore, PC films showed high optical absorption in ultraviolet (UV) and near-infrared (NIR) regions.^45,46^ Despite the complex chemical composition of PC, it has been shown from an X-ray diffraction analysis that the PC films could have crystal or amorphous structures according to the used substrate type.^46−48^ The temperature dependence of the PC conductivity revealed a semiconductor behavior in the temperature range 283–300 K.^44^

To summarize, a great deal of research on the PC exists in the pharmaceutical, medicinal, and chemical fields. On the other hand, very few works deal with the physical properties and potential technical applications of propolis as a natural material or as a mixture with other materials. To unravel the optical and electrical properties of PC and shed light on potential technical applications of PC, we have studied thin films of PC.

The aim of our study is to determine the morphological, optical, and electrical properties of the PC film and to reveal the high potential of this biological organic material for technical applications.

The layout of this paper is as follows: first, we describe the experimental techniques used for the elaboration and for the characterization of the propolis films; second, we present morphological, optical, and electrical results and discuss them; and then, we summarize our findings in a conclusion.

## 2 Experimental Section

### 2.1 Raw Materials

Raw PC was produced by *Apis mellifera* bee species on the island of Djerba in Tunisia. For the PC extract solution, we used ethanol (99.9% vol) as a solvent. PC films were deposited on quartz and amorphous glass substrates for the optical characterization and on tin oxide (SnO_2_) substrates for electric characterization. Silver was used for electrical contacts.

### 2.2 Methods

For the ethanolic extract solution preparation, the PC sample was used at room temperature ranging between 20 and 25 °C. A quantity of 10 g of raw PC was macerated in 30 mL of solvent (ethanol 99.9%). The PC extract solution was obtained after 7 days of maceration. We have noticed that this PC extract solution is very stable. The stability of the PC extract solution was deduced from the fact that layers of propolis produced by the same initial PC solution at different times (more than one month delay) exhibited similar optical and electrical properties. Furthermore, the appearance color of the extract solution does not change over time. To prepare PC films, a few drops of ethanolic extract was deposited on the heated substrates until the alcohol completely evaporated (drop-casting technique). The substrate temperature was varied from 40 to 120 °C. No effect of the preparation temperature has been observed on the optical and electrical properties of the PC films. Moreover, the produced thin layers exhibit good performance stability. The PC film stability was deduced from the fact that the optical and electrical measurements on the same layer, at time intervals of several weeks, gave similar results. The stability of any layer and its resistance to aging is a remarkable quality necessary for any application. A follow-up of the optical and electrical properties of the PC layer over several months should be the subject of more detailed research. On the other hand, we have tried to dissolve the raw propolis in distilled water, but the solubility is very poor, even after several weeks of maceration. Hence, the hydrophobic behavior of propolis should be expected. Furthermore, when we put a water drop on the PC film, it keeps a spherical shape. So, the propolis film displays hydrophobic behavior. Nevertheless, this needs more investigation to be stated with more confidence.

Optical measurements were performed using a Shimadzu UV-3101 PC spectrophotometer. Electrical studies were carried out using an Agilent 4294 A impedance analyzer. Current–voltage characteristics were measured using a Keithley 220 current generator and an Agilent 34000 multimeter. Surface morphology of the PC films was explored by atomic force microscopy (AFM) and scanning electron microscopy (SEM). AFM and SEM equipments are an XE-100 (Park Systems Corporation) in noncontact mode (NC-AFM) and a Philips FEG-XL30s operating at 3 kV with an FEI Quanta 400 FEG ESEM operating at 10 kV, respectively.

## 3 Results and Discussion

### 3.1 Morphological Study

**Figure 1:**
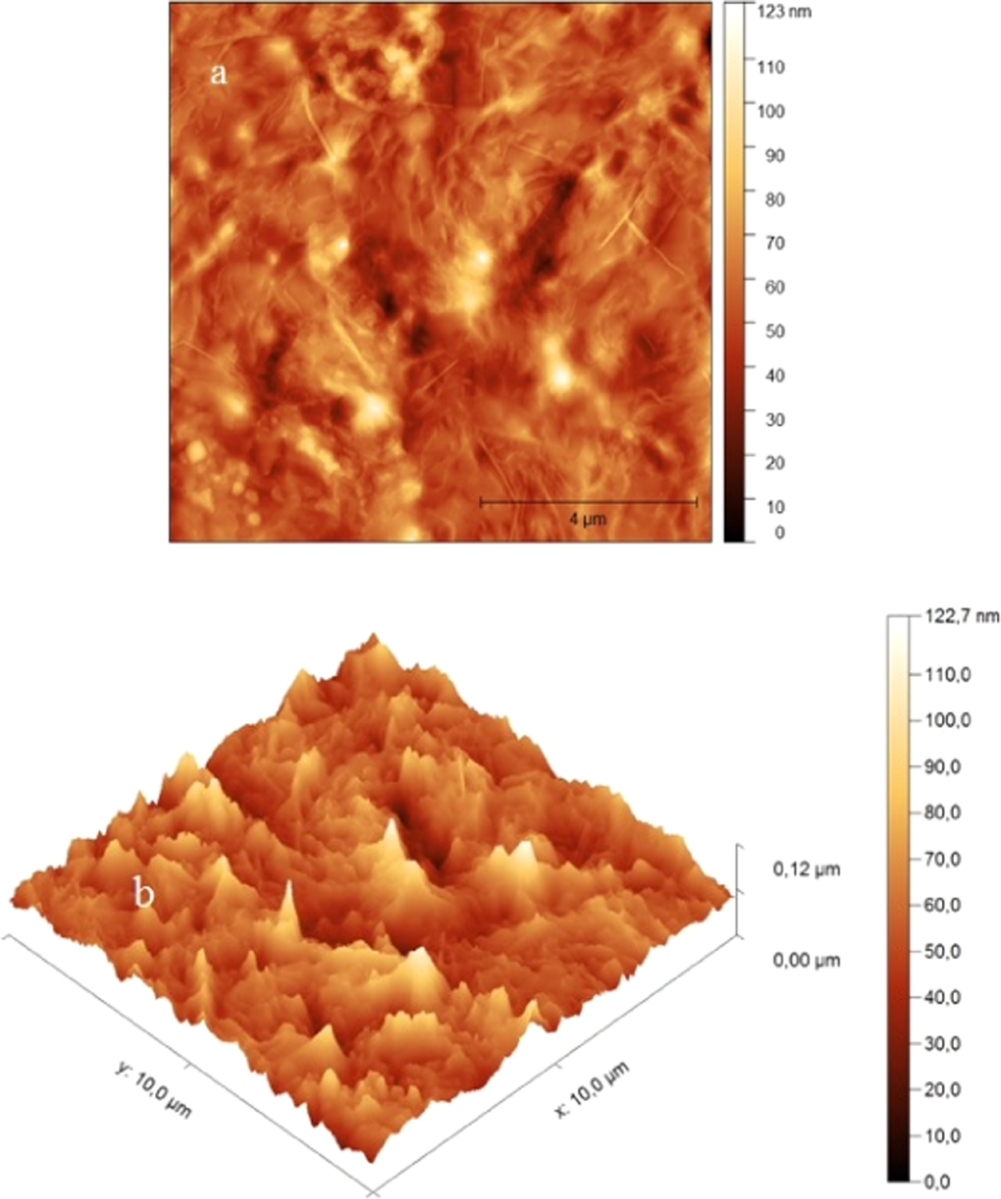
2D (a) and 3D (b) AFM images of the PC film.

Atomic force microscopy (AFM) topography of our PC thin-film sample is shown in Figure 1. The PC sample surface appears dense, well covered, without cracks, and on which small peaks are noticeable. An area of 100 μm^2^ of the PC film was used to calculate the root-mean-square (RMS) roughness value. It is found to be around 11 nm. This low RMS roughness value indicates that the surface of the PC film is smooth and adequate for optical applications.

**Figure 2:**
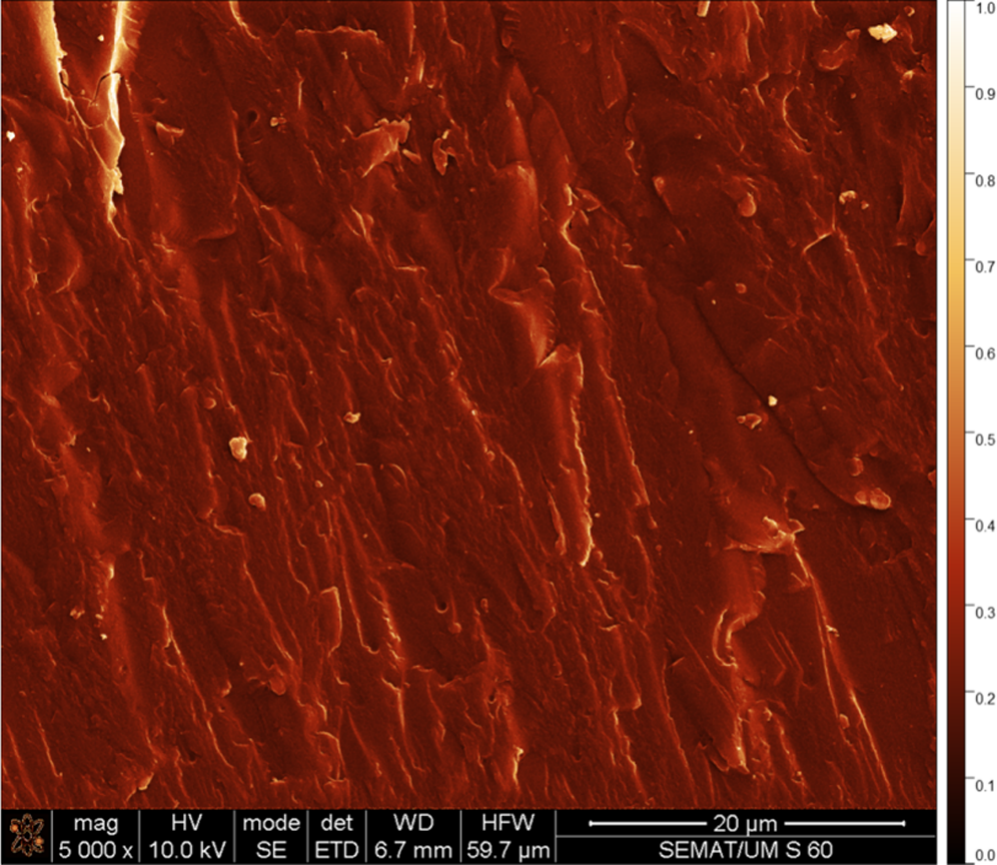
Cross-sectional SEM image of the PC film.

The scanning electron microscopy (SEM) image of the PC sample is displayed in Figure 2. SEM analysis of the PC sample is carried out at a magnification of 5000 times. This cross-sectional SEM image reveals a well-covered surface without pinholes or fissures. The PC film appears dense and continuous. This confirms the effectiveness of the used growth technique of the PC layer despite its simplicity. The layer thicknesses, given by the cross-sectional SEM images, were 52.3, 43.6, and 32.2 μm for the three tested PC films.

### 3.2 Optical Properties

**Figure 3:**
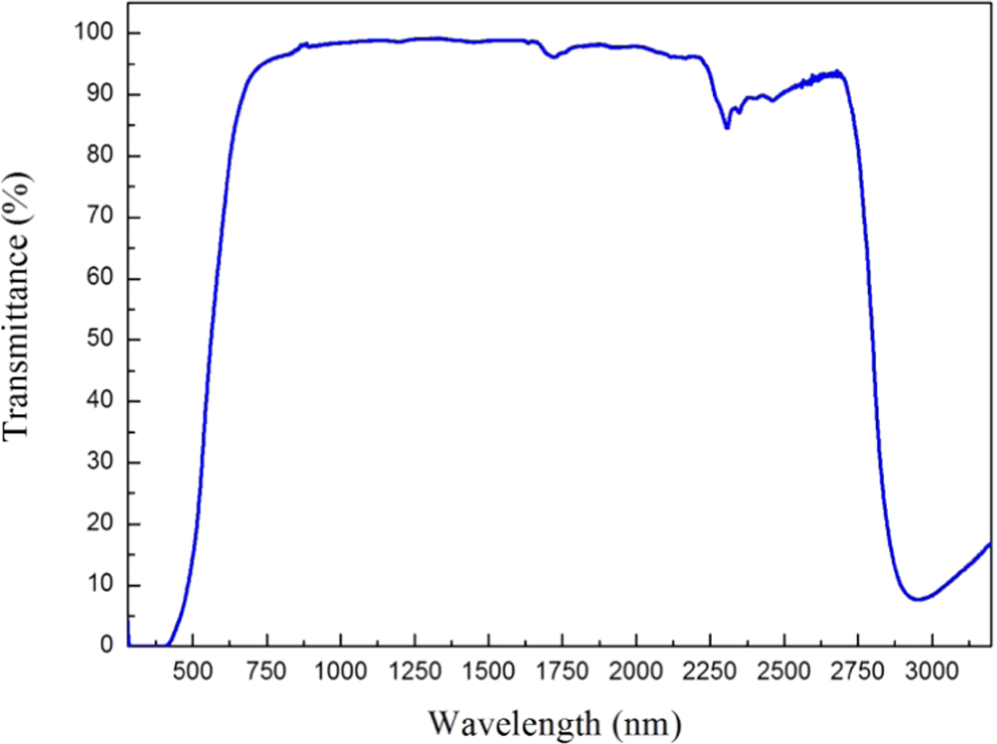
Transmission spectrum of the PC film deposited on amorphous glass. The spectrum is measured at ambient temperature (∼300 K).

Optical properties were investigated using UV–visible–NIR spectroscopy. Figure 3 shows the transmittance spectrum of the PC film deposited on an amorphous glass substrate. As we can see from the transmittance spectra, for UV radiation and the blue components of visible light, the PC film is opaque (wavelength λ < 500 nm). So, the PC film can be used as a UV barrier and a blue light filter. For green and yellow light (∼500–600 nm), which corresponds to the maximum of solar irradiation spectra, the PC film exhibits high absorption and the absorption coefficient abruptly changes. This rapid variation corresponds to a fundamental absorption edge, typical of semiconductors. However, for red and near-infrared radiation (∼600–2700 nm), the PC film is transparent (transmittance > 95%). Then, in the 2700–3200 nm wavelength range, absorption increases sharply. This last behavior is attributed to molecular vibration. In this last spectral region, corresponding to the frontier between NIR and mid-infrared, PC films reveal significant photosensitivity and can be used as photosensors. The weak absorption peaks observed at λ = (1725; 2306; 2462) nm correspond to the molecular vibration of low-concentration molecules in raw propolis. We obtained the same optical behavior for the PC film deposited on a quartz substrate. This confirms that the absorption increase beyond 2700 nm is related to the PC layer and not to the substrate. In light of the transmittance spectra, the PC film seems like a bandpass filter. This filter cuts off UV radiation, which is responsible for the oxidation of foods, leading to nutrient losses, degradation of flavors, and discoloration. In harmony with these findings and in a recent medical study, Kim et al. discovered that propolis reveals protective effects against skin aging induced by UV light.^49^ Furthermore, in a new study, the incorporation of the propolis extract into pectin improved the UV-light barrier properties of the pectin films.^50^ Thus, the PC film can be used in food packaging to prevent food deterioration. Ulloa et al. published the Fourier transform infrared (FTIR) spectroscopy spectra of raw propolis and ethanolic extract of propolis, proving the presence of aromatic rings, flavonoids, and other functional groups in propolis.^38^ These compounds have benzoic cycles. The benzoic structure exhibits high resonance regarding UV radiation,^51^ which may explain the UV-blocking phenomenon of PC. The propolis film is also a good bandpass filter for the range 600−2700 nm. Therefore, the PC film can be used as a filter for optical instruments and setups.

**Figure 4:**
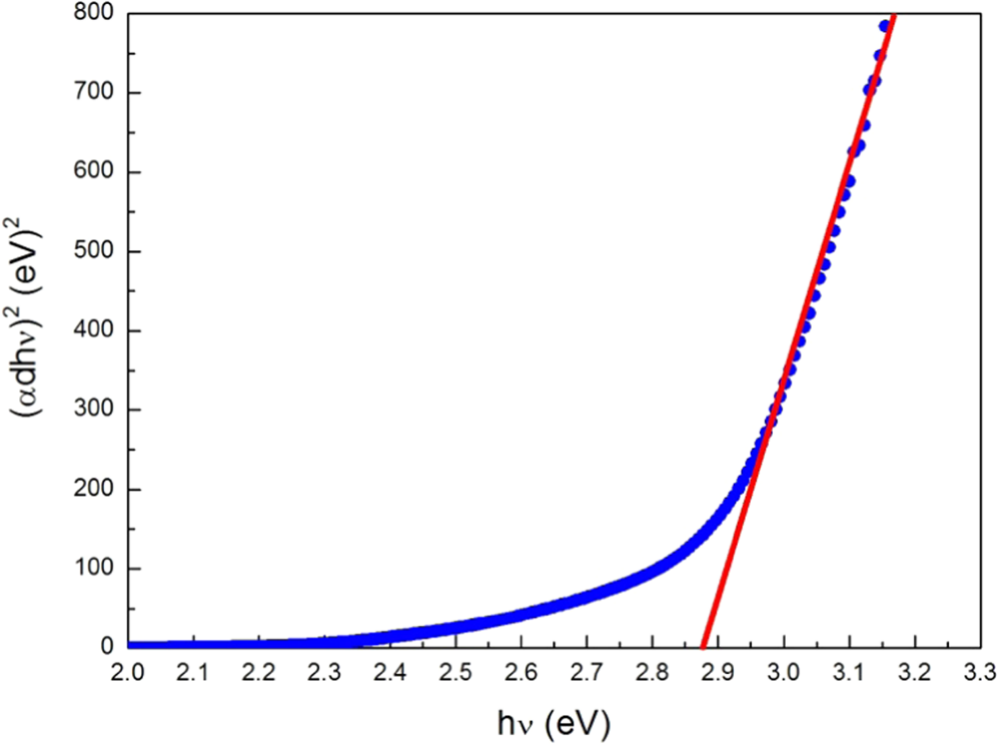
Plot of (α*dh*ν)^2^ versus photon energy (*h*ν): energy gap estimation, experiment (full circles), and extrapolation of absorption edge (full line).

The low-wavelength edge of transmission spectra (Figure 3) reveals rapid absorption variations corresponding to an energy gap *E*_*g*_ (similar to the optical band gap in inorganic semiconductors). *E*_*g*_ was determined from the (α*dh*υ)^2^ versus photon energy (*h*υ) plot (Figure 4, which is typical of direct optical transition), where α is the absorption coefficient, *d* is the film thickness, and *h* is Planck’s constant. The value of the *E*_*g*_ was extracted by the Tauc formula to fit the linear region of the (α*dh*υ)^2^ versus (*h*υ) plot corresponding to the absorption edge.^52−55^ The PC film energy gap was estimated *E*_*g*_ ≈ 2.88 eV at room temperature (∼300 K), which in principle enables optoelectronic applications in the UV and blue ranges. These results agree with the experimental findings of Drapak et al.^44−46^ obtained from transmittance (3.07 eV) and photoluminescence spectra (2.86 eV) of Ukrainian propolis films. The *E*_*g*_ ≈ 2.88 eV corresponds to a wavelength λ = 430 nm, almost equal to the blue light wavelength (405 nm) used to read and record information in blue-ray and high-density digital versatile disc (HD DVD) optical devices.

PC films show optical properties quite similar to those of zinc oxide (ZnO) thin films: high transmittance in the visible range, high absorption in UV light, ZnO band gap of almost 3.4 eV, antimicrobial properties, and low toxicity to the human body.^42,56−58^ Therefore, propolis films can be viewed as a low-cost and green potential alternative to ZnO in optical and electronic devices at low temperatures.

Thus, in light of the results concerning the optical properties of propolis films displayed above, this natural and biologically active material presents several potential applications in short-wavelength optoelectronics (photodetectors, light-emitting diodes that operate in blue and UV ranges, etc.) and transparent electronics.

### 3.3 Electrical Properties

**Figure 5:**
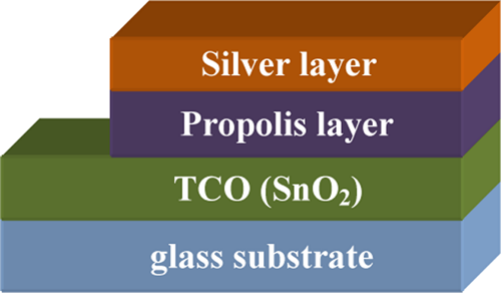
Schematic illustration of the structure used in electrical investigation.

The electrical properties of PC film samples were investigated using the structure displayed in Figure 5. This structure is composed of several layers deposited on an amorphous glass substrate with the following sequence: tin oxide (SnO_2_), which is a transparent conducting oxide (TCO), PC film, and silver layer. SnO_2_ and the silver layer play the role of Ohmic contacts.

#### 3.3.1 Conductance Results

**Figure 6:**
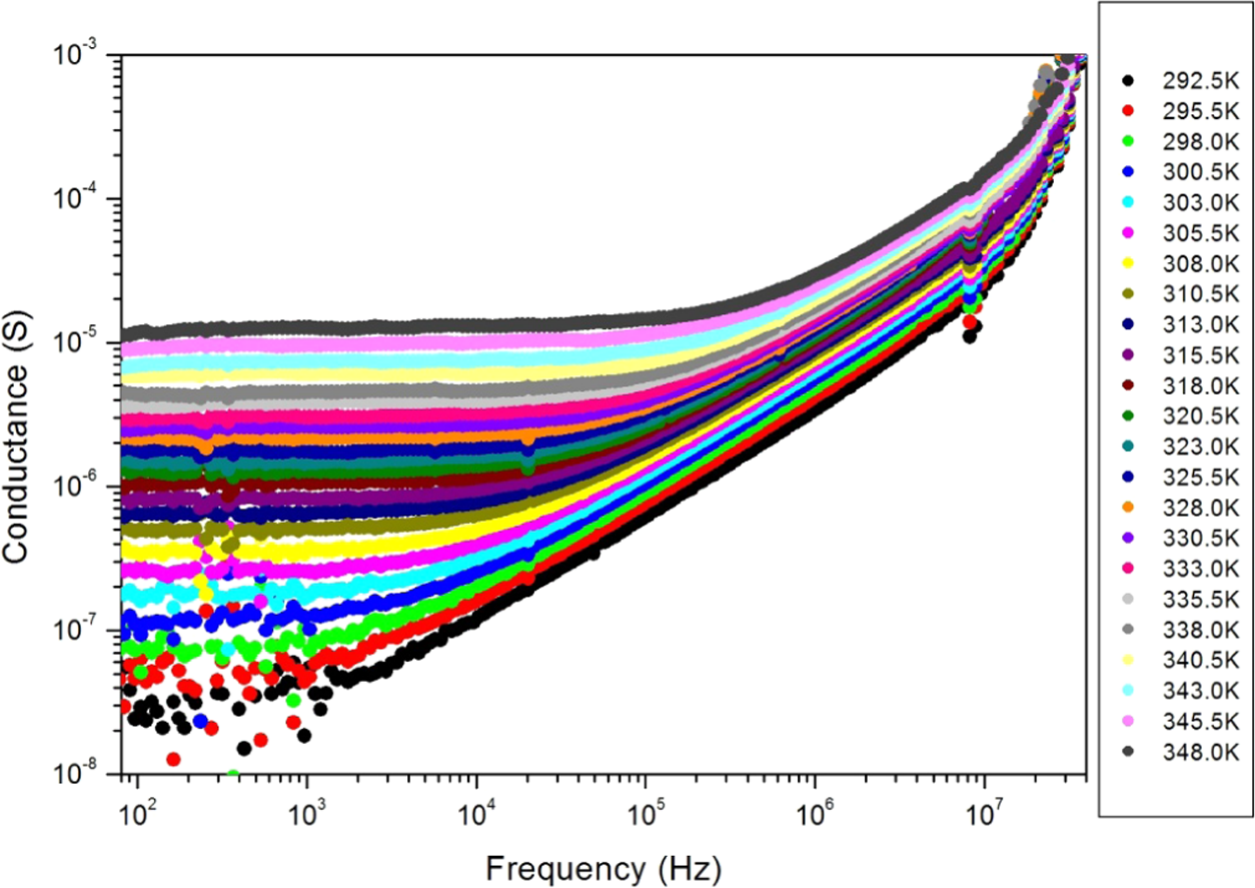
AC conductance versus frequency at several temperatures of the propolis film.

Electrical conductance constitutes the first-rank tool in material characterization. AC complex impedance spectroscopy was used to extract the electrical properties of PC films for a large frequency range (40 Hz–40 MHz), at different temperatures (292–348 K), and in ambient air. The obtained results are well reproducible. Figure 6 displays the AC conductance in siemens (*S*) versus frequency (*f*) at several PC film absolute temperatures (*T*). The conductance curve presents two regions: a frequency-independent region (plateau) observed along a low-frequency band and a frequency-dependent region at higher frequencies. The frequency band of the plateau enlarges with temperature, which reflects the decrease of the relaxation time of the sample with temperature. This is due to the release of charge carriers by thermal energy. The electrical conductance increases with temperature and with frequency also, which is a sign of semiconductor behavior. At the low-frequency range, we observe a large variation in the conductance (10^–8^–10^–5^ S) for a relatively small variation of temperature (292–348 K). So, the PC film can have a potential application as a safe and biocompatible negative-temperature-coefficient sensor, a current-limiting device, and a thermal threshold controller in bioelectronics. These latter properties are very interesting since they are observed at temperatures quite close to the ambient temperatures that one might have throughout the year. This is important for applications operating at ambient temperatures.

**Figure 7:**
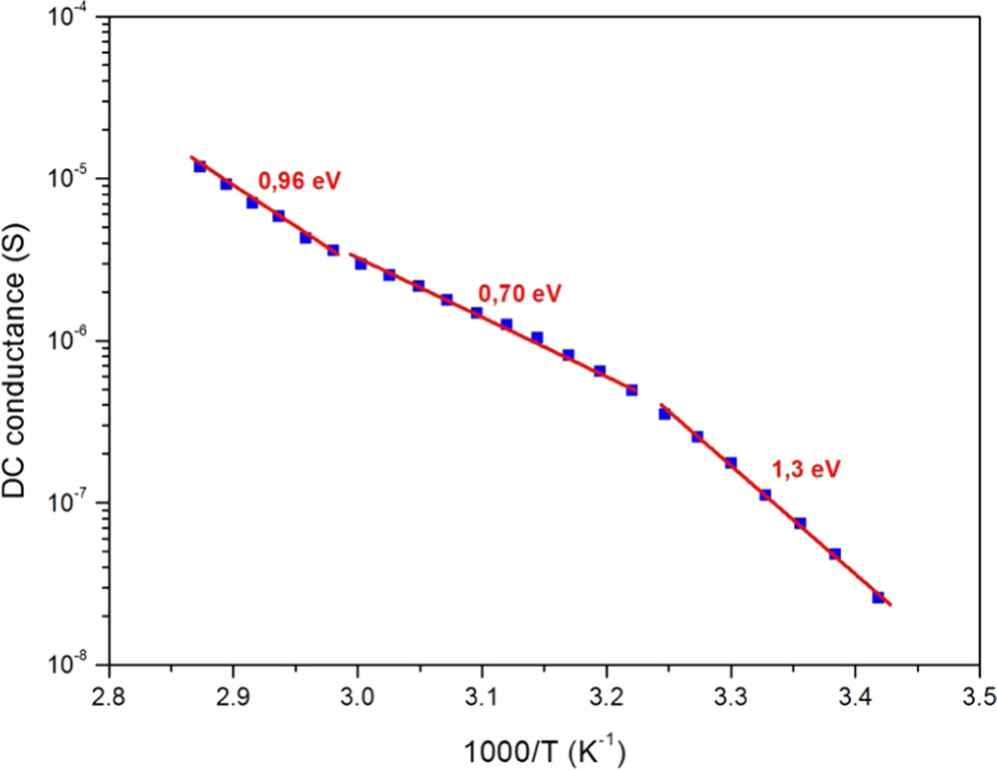
Experimental DC conductance (full squares) versus 1000/*T* and its linear fit (full lines) with the activation energy values.

From the low-frequency plateau, we extracted the “DC” regime conductance or frequency-independent conductance (*G*_DC_). Figure 7 shows the *G*_DC_ evolution as a function of 1000/*T* in an Arrhenius plot. In Figure 7, we can see three linear slopes for our experiment’s temperature range. These linear curves reveal thermally activated processes. Thus, *G*_DC_ can be described by an Arrhenius behavior^59^
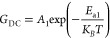
1where *A*_1_ is a pre-exponential factor, *E*_a1_ is the thermal activation energy, *K*_*B*_ is the Boltzmann constant, and *T* denotes the PC film’s absolute temperature. The three slopes give three activation energy values given in Figure 7 and summarized in Table 4. These different activation energies correspond to several emission centers of charge carrier and represent a sign of the coexistence of various conduction mechanisms and charge carriers (electronic conduction, hole conduction, ionic vacancy conduction, ionic conduction, etc.).^59−61^

In the AC regime, a simple single power-law conductance behavior is widely observed in diverse materials of physical and chemical different properties. This universal power law was given by Jonscher^62−65^
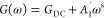
2where *G*_DC_ is the DC conductance, *A*_1_^′^ is a temperature-dependent factor, ω is the angular frequency, and *s* is a power exponent dependent on temperature and ranging from 0 to 1. However, all materials do not conform to this simple behavior. Double power-law terms^66^ are sometimes required to describe the AC response. Moreover, conduction mechanisms other than hopping can occur at different frequencies, such as the Drude conduction mechanism or the percolation one. Below *f* ≲ 8 × 10^6^ Hz, the experimental data are well fitted by eq 2. The fitting parameters values of *G*_DC_, *A*′_1_, and *s* are presented in Table 1.

**Table 1:** “DC” Regime Conductance (*G*_DC_) and the Fitting Parameters Values for the Power-Low Function with Their Standard Deviation Errors

*T* (K)	*G*_DC_ (*S*)	*A*′_1_(10^–10^ S Hz ^–s^)	*s*
292.5	(2.78 ± 0.36)10^–8^	(0.71 ± 0.03)	(0.780 ± 0.003)
295.5	(4.67 ± 0.40)10^–8^	(0.98 ± 0.03)	(0.772 ± 0.002)
298	(7.15 ± 0.59)10^–8^	(0.98 ± 0.04)	(0.782 ± 0.003)
300.5	(1.06 ± 0.04)10^–7^	(1.24 ± 0.03)	(0.774 ± 0.002)
303	(1.57 ± 0.04)10^–7^	(1.35 ± 0.04)	(0.776 ± 0.002)
305.5	(2.46 ± 0.09)10^–7^	(1.28 ± 0.04)	(0.787 ± 0.002)
308	(3.32 ± 0.08)10^–7^	(1.29 ± 0.04)	(0.793 ± 0.002)
310.5	(4.60 ± 0.06)10^–7^	(1.38 ± 0.03)	(0. 793± 0.002)
313	(6.12 ± 0.09)10^–7^	(1.28 ± 0.03)	(0.802 ± 0.002)
315.5	(7.66 ± 0.09)10^–7^	(1.36 ± 0.03)	(0.802 ± 0.002)
318	(9.92 ± 0.09)10^–7^	(1.13 ± 0.04)	(0.821 ± 0.002)
320.5	(1.22 ± 0.02)10^–6^	(0.94 ± 0.04)	(0.836 ± 0.003)
323	(1.41 ± 0.01)10^–6^	(1.06 ± 0.03)	(0.831 ± 0.002)
325.5	(1.69 ± 0.02)10^–6^	(0.93 ± 0.03)	(0.843 ± 0.002)
328	(2.06 ± 0.01)10^–6^	(1.06 ± 0.04)	(0.837 ± 0.003)
330.5	(2.44 ± 0.02)10^–6^	(1.18 ± 0.04)	(0.831 ± 0.002)
333	(2.91 ± 0.02)10^–6^	(1.17 ± 0.04)	(0.836 ± 0.002)
335.5	(3.67 ± 0.02)10^–6^	(1.03 ± 0.04)	(0.849 ± 0.003)
338	(4.35 ± 0.02)10^–6^	(0.72 ± 0.03)	(0.878 ± 0.003)
340.5	(5.84 ± 0.03)10^–6^	(0.55 ± 0.03)	(0.900 ± 0.004)
343	(7.15 ± 0.03)10^–6^	(0.63 ± 0.04)	(0.894 ± 0.004)
345.5	(9.51 ± 0.03)10^–6^	(0.50 ± 0.03)	(0.914 ± 0.003)
348	(1.24 ± 0.01)10^–5^	(0.55 ± 0.03)	(0.913 ± 0.004)

The *s* exponent is almost equal to 0.8 and temperature-independent. Thus, this behavior indicates that the transport mechanism, in AC conduction, mostly occurs by the quantum mechanical tunneling process.^67^

The conductance spectrum exhibits a sharp discontinuity around 8 × 10^6^ Hz and a rapid increase in conductance thereafter. This behavior is typical of the percolation mechanism. Probably, such a frequency corresponds to the frequency emission of a specific trap in the PC sample. This leads to an increase in the density of the free charge carriers and, consequently, to a rapid increase in the conductance.

**Figure 8:**
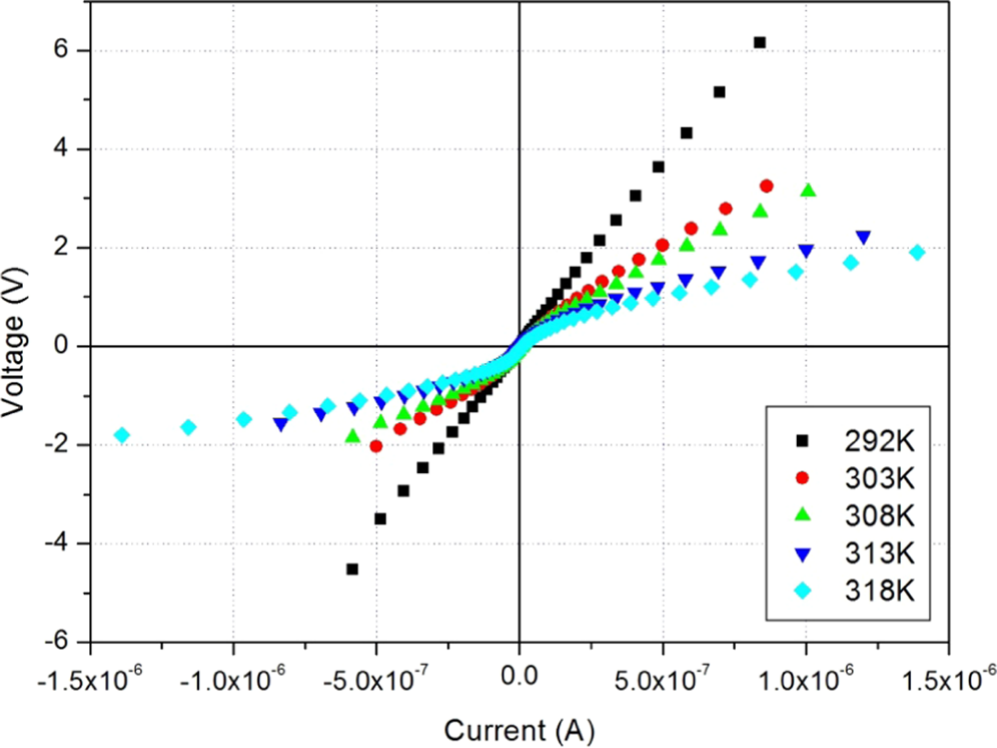
Static characteristics (voltage versus applied current) of the PC sample for different PC film temperatures.

The static characteristics (voltage versus applied current) of the PC film were determined (see Figure 8) for different PC film temperatures (292–318 K). The characteristic curves are symmetrical with respect to the zero value of the potential and do not show an open-circuit voltage. This confirms that the metal/PC contacts are of Ohmic type. These characteristics show linear behavior within our measurement voltage range (−6 to +6 v) with temperature-dependent behavior. This confirms that different types of charge carriers contribute to the conduction process.

The slopes of the linear part of these curves give access to the sample conductance *G*_s_ in the static regime for several temperatures. The *G*_s_ results are displayed in Table 2. These results are quite similar to the DC conductance values obtained above (see Table 1), which proves that the conductance, obtained at the low-frequency regime, corresponds effectively to the DC conductance of the PC sample.

**Table 2:** PC Film Conductance *G*_s_ in the Static Regime for Several PC Film Temperatures

temperature (K)	*G*_s_(10^–7^ S)
292	(1.35 ± 0.01)
303	(2.48 ± 0.04)
308	(3.12 ± 0.06)
313	(5.92 ± 0.19)
318	(9.03 ± 0.31)

#### 3.3.2 Impedance Measurements

Impedance spectroscopy is a powerful, nondestructive method used to investigate the electrical and electrochemical behavior of different materials and systems. The real part *Z*′ and imaginary part *Z*″ of the PC sample complex impedance *Z* were measured for a large frequency range (40 Hz–40 MHz), at different temperatures (292–348 K), and in ambient air.
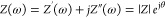
3where *j*^2^ = −1, ω is the angular frequency, θ is the impedance phase angle corresponding to the phase difference between the voltage and the current signals, and |*Z*| is the magnitude of the impedance. The real part *Z*′ quantifies the resistance to the current flow (Ohmic effect), and the imaginary part *Z*″ characterizes the opposition to the fluctuation of voltage or current flow (capacitive and inductive effect).

**Figure 9:**
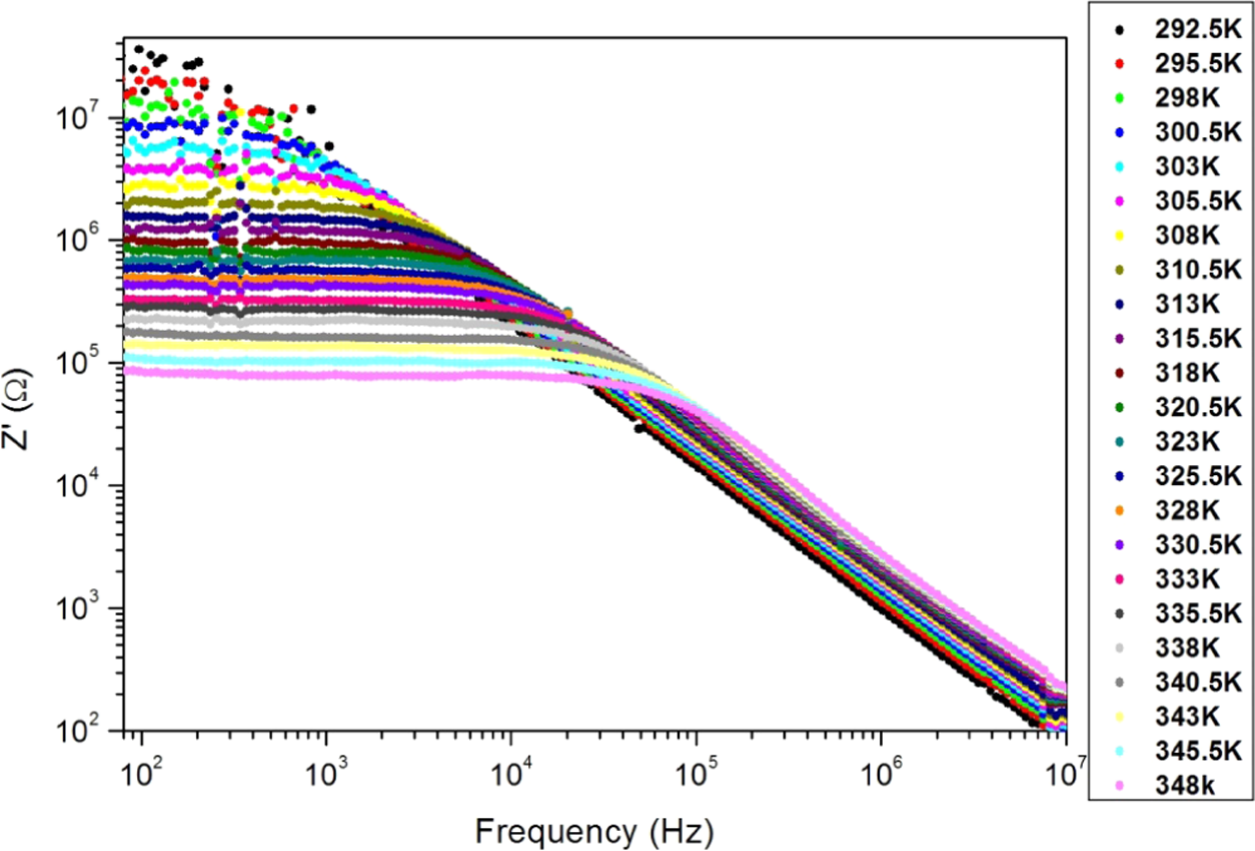
Frequency dependence of the impedance real part of the PC sample for different PC film temperatures.

Figure 9 displays the frequency dependence of *Z*′ for different measurement temperatures. We note that *Z*′ decreases with an increase of the testing frequency and temperature. Therefore, PC samples reveal semiconductor behavior. We observe a frequency-independent region (plateau) along a low-frequency band. The frequency band of the plateau expands with temperature, which reflects the decrease in the relaxation time of the sample with temperature. This is due to the release of charge carriers by thermal energy. However, *Z*′ decreases sharply at high frequencies and merges for all sample temperatures, which proves the presence of polarization processes. In Table 3, we report the average (*Z*′_moy_) of *Z*′ values corresponding to the plateau.

**Table 3:** Average (*Z*′_moy_) of *Z*′ Values Corresponding to the Plateau with Their Standard Deviation Errors, the Plateau Upper-Limit Frequencies (*f*_c_), and the Relaxation Frequencies (*f*_r_) for Different PC Film Temperatures

temperature (K)	*Z*′_moy_ (Ω)	*f*_c_ (Hz)	*f*_r_ (Hz)
292.5	(2.68 ± 0.74)10^7^	220	
295.5	(1.76 ± 0.38)10^7^	318	577
298	(1.32 ± 0.28)10^7^	497	776
300.5	(8.38 ± 0.76)10^6^	721	1124
303	(5.68 ± 0.42)10^6^	900	1513
305.5	(3.76 ± 0.36)10^6^	1513	2191
308	(2.75 ± 0.21)10^6^	2035	2948
310.5	(2.00 ± 0.16)10^6^	2737	4270
313	(1.51 ± 0.07)10^6^	3682	5334
315.5	(1.24 ± 0.08)10^6^	4270	6662
318	(0.95 ± 0.06)10^6^	5334	8962
320.5	(0.80 ± 0.04)10^6^	7175	10 390
323	(0.68 ± 0.03)10^6^	7727	12 060
325.5	(0.57 ± 0.03)10^6^	8962	15 060
328	(0.48 ± 0.02)10^6^	11 190	17 470
330.5	(0.42 ± 0.01)10^6^	12 980	20 260
333	(3.22 ± 0.06)10^5^	16 220	27 250
335.5	(2.77 ± 0.09)10^5^	17 470	34 040
338	(2.21 ± 0.05)10^5^	23 490	42 520
340.5	(1.69 ± 0.05)10^5^	29 350	53 110
343	(1.39 ± 0.02)10^5^	31 610	66 330
345.5	(1.03 ± 0.01)10^5^	45 790	82 850
348	(0.79 ± 0.02)10^5^	61 590	103 500

**Figure 10:**
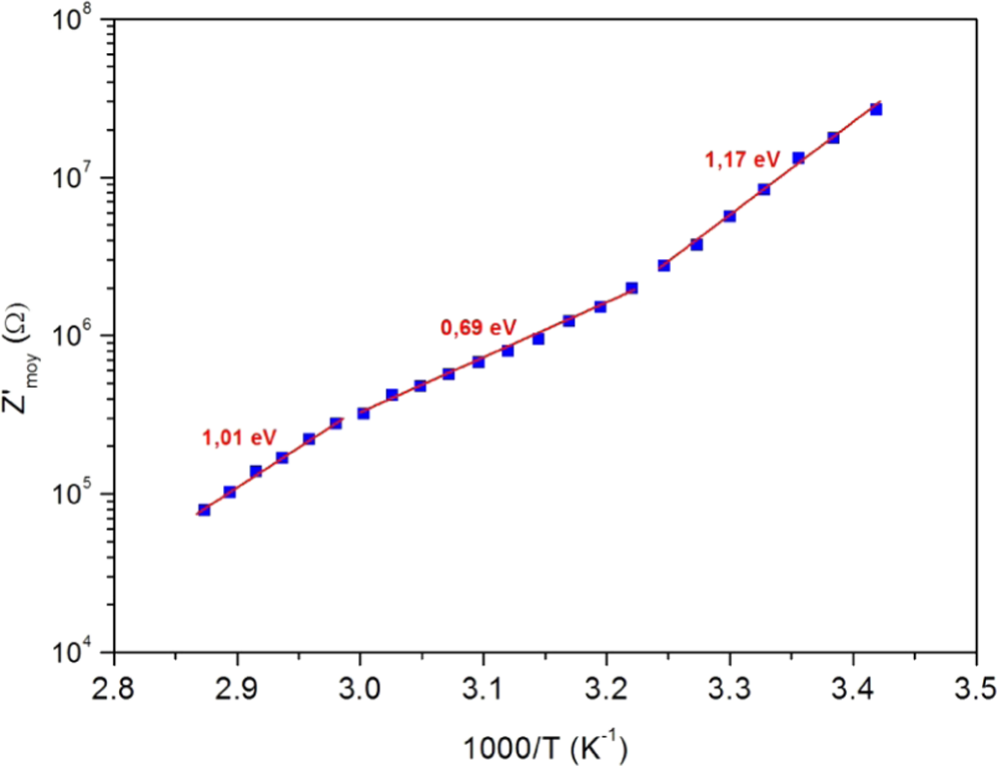
Average (*Z*′_moy_) of *Z*′ values corresponding to the plateau (full squares) versus 1000/*T* and its linear fit (full lines) with the activation energy values.

Figure 10 shows the *Z*′_moy_ evolution as a function of 1000/*T* on a semilog scale. In this last figure, we can see three linear slopes for our experiment’s temperature range, which reveals thermally activated processes. Thus, *Z*′_moy_ can be described by an Arrhenius behavior^59^
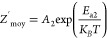
4where *A*_2_ is a pre-exponential factor, *E*_a2_ is the activation energy, *K*_*B*_ is the Boltzmann constant, and *T* denotes the PC film absolute temperature. The three slopes give three activation energy values summarized in Table 4. These different activation energies correspond to several relaxation processes and confirm the coexistence of various conduction mechanisms and charge carriers.^59−61^ The activation energy values obtained using *Z*′_moy_ are very close to those obtained using DC conductance.

**Table 4:** Activation Energies (*E*_a1_), (*E*_a2_), (*E*_a3_), and (*E*_a4_) Obtained Respectively from the Arrhenius Plot of *G*_DC_, *Z*′_moy_, *f*_r_, and *f*_c_ with Their Corresponding Temperature Ranges

temperature range (K)	*E*_a1_ (eV)	*E*_a2_ (eV)	*E*_a3_ (eV)	*E*_a4_ (eV)
292–309	1.30 ± 0.03	1.17 ± 0.03	1.05 ± 0.02	1.26 ± 0.03
309–334	0.70 ± 0.02	0.69 ± 0.02	0.67 ± 0.03	0.70 ± 0.02
334–348	0.96 ± 0.03	1.01 ± 0.03	0.89 ± 0.01	0.99 ± 0.03

**Figure 11:**
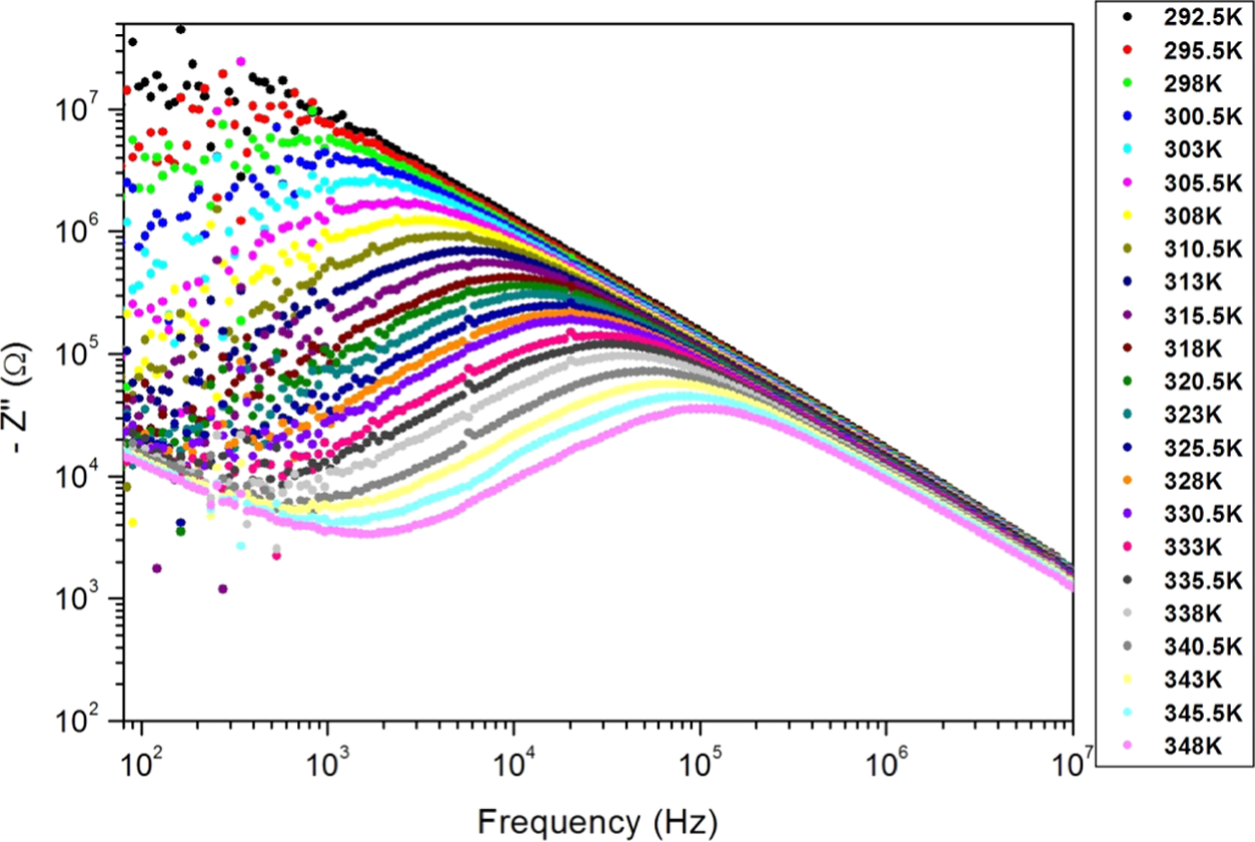
Frequency dependence of the impedance imaginary part (*Z*″) of the PC sample for different PC film temperatures.

The frequency dependence of the imaginary part *Z*″ of the complex impedance of the PC sample for different temperatures is displayed in Figure 11. We observe that *Z*″ spectra show the appearance of peaks at specific frequencies *f*_r_ (displayed in Table 3), which confirms the presence of polarization phenomena. The peaks’ frequencies correspond to the relaxation frequencies for different temperatures. As shown in Figure 11, the relaxation frequency increases with temperature, which is due to the enhancement of charge carrier mobility. We notice that the peak height decreases with temperature, a sign of the decrease of resistive properties.^68^ These results confirm the semiconductor behavior of our PC sample.^69^ The relaxation time τ_r_ associated with the *f*_r_ frequency is given by
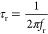
5However, *Z*″ decreases sharply at high frequencies and merges for all sample temperatures. At high frequencies, the relaxation time becomes smaller and smaller. Thereby, the polarization phenomenon vanishes at high frequencies.^70^

**Figure 12:**
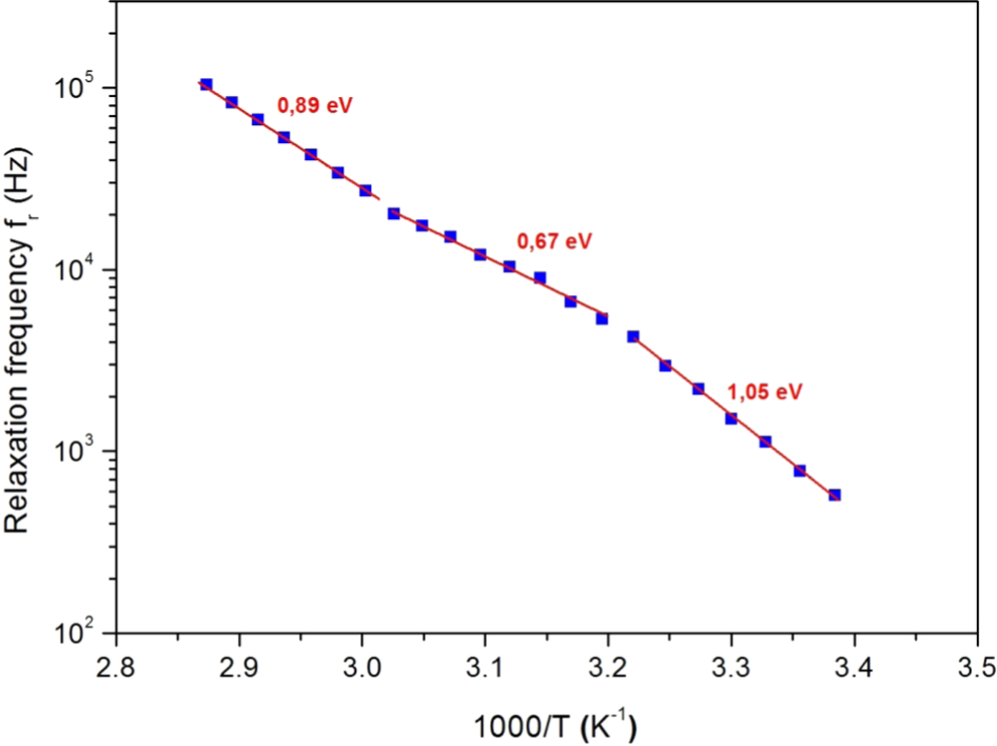
Relaxation frequency (*f*_r_) (full squares) versus 1000/*T* and its linear fit (full lines) with the activation energy values.

Figure 12 displays the relaxation frequency *f*_r_ as a function of 1000/*T* on a semilog scale. In this figure, we can also distinguish three linear slopes for the experiment’s temperature range, which proves the presence of thermally activated processes. Therefore, *f*_r_ can be described by an Arrhenius behavior^59^
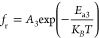
6

From the three slopes of Figure 12, we obtained three activation energy values, which are recapitulated in Table 4. These activation energies correspond to several relaxation processes and prove the coexistence of various relaxation mechanisms.^59−61^ The activation energy values obtained using *f*_r_ are very close to those obtained using *Z*′_moy_ and the DC conductance. This attests that the electrical polarization and conductance processes have the same origin.

**Figure 13:**
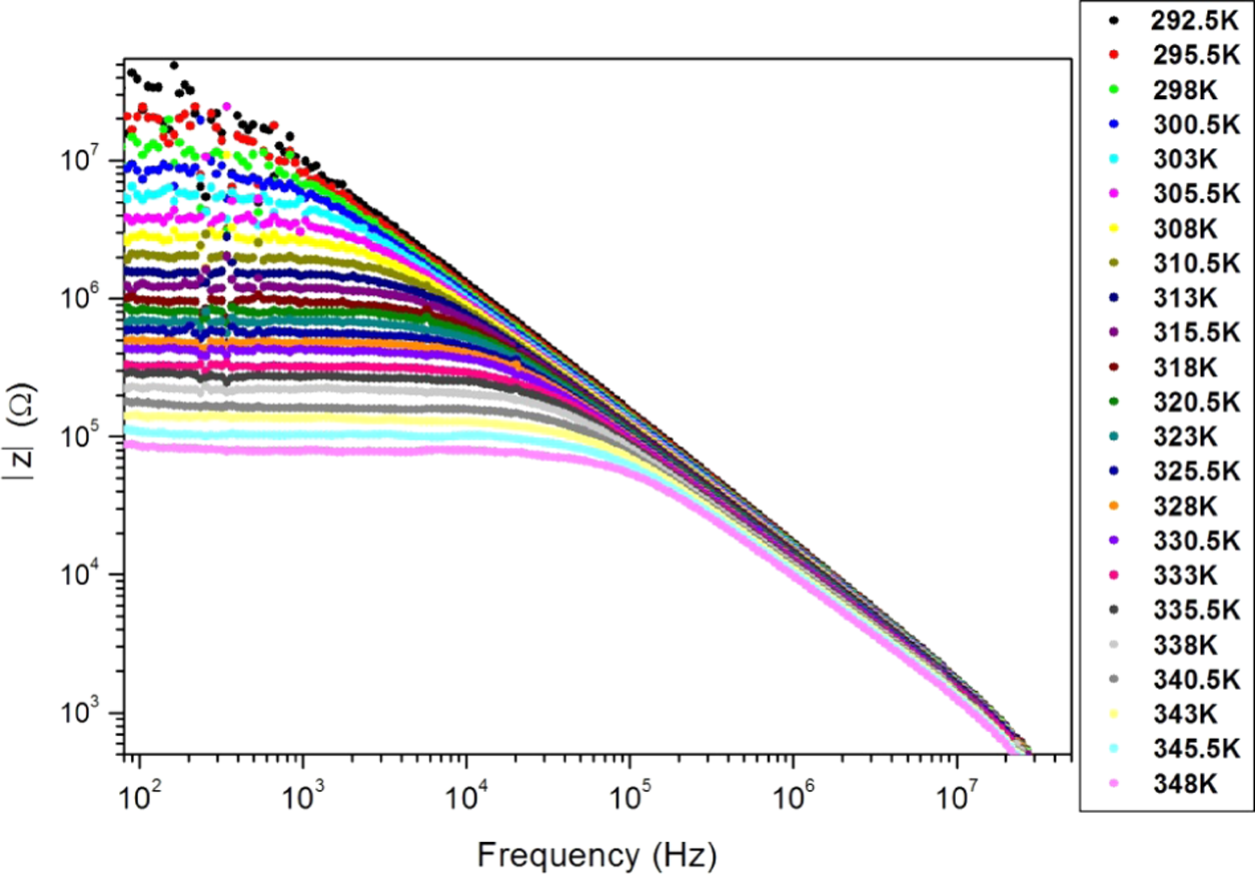
Impedance magnitude (|*Z*|) versus frequency on a logarithmic scale (Bode diagram) for different PC film temperatures.

The impedance magnitude |*Z*| of the PC sample is displayed in Figure 13 as a function of the frequency for different measurement temperatures. |*Z*| decreases with an increase in frequency and temperature. We discern a frequency-independent region (plateau) along a low-frequency band. The frequency band of the plateau expands with temperature, which reveals the decrease of sample relaxation time with temperature. This is due to the release of charge carriers by thermal energy. On the other hand, |*Z*| decreases sharply at high frequencies and merges for all sample temperatures, which proves the presence of relaxation processes. In Table 3, we report the upper-limit frequency (*f*_c_) of the |*Z*|′s plateau for all measurement temperatures. This upper-limit frequency corresponds to |*Z*|_moy_/√2, where |*Z*|_moy_ is the average of the impedance magnitude |*Z*| for the plateau values.

**Figure 14:**
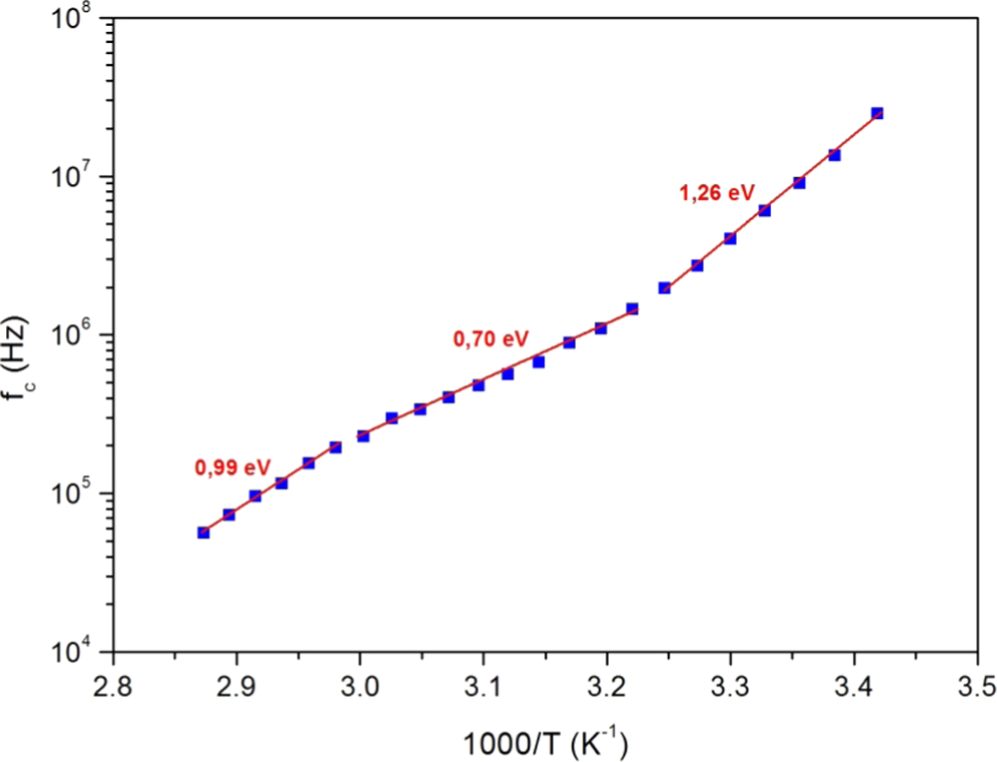
Upper-limit frequency (*f*_c_) (full squares) versus 1000/*T* and its linear fit (full lines) with the activation energy values.

The upper-limit frequency (*f*_c_) versus 1000/*T* is displayed in Figure 14 on a semilog scale. In Figure 14, we discern three linear slopes for our experiment’s temperature range. This proves the presence of thermally activated processes corresponding to different phase transitions. *f*_c_ can then be described by an Arrhenius behavior^59^
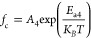
7

From the three slopes in Figure 14, we obtained three activation energy values, which are presented in Table 4. These activation energies confirm the coexistence of various relaxation mechanisms.^59−61^ The activation energy values obtained using *f*_c_ are very close to those obtained using relaxation frequency *f*_r_, *Z*′_moy_, and DC conductance.

**Figure 15:**
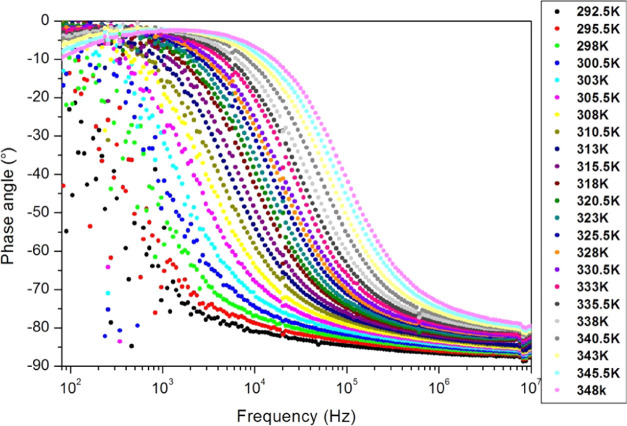
Impedance’s phase angle versus frequency of the PC’s film on a semilogarithmic scale (Bode diagram) for different temperatures.

The impedance’s phase angle θ, corresponding to the phase difference between the voltage and the current signals at different temperatures, is plotted in Figure 15 on a semilog scale.

**Figure 16:**
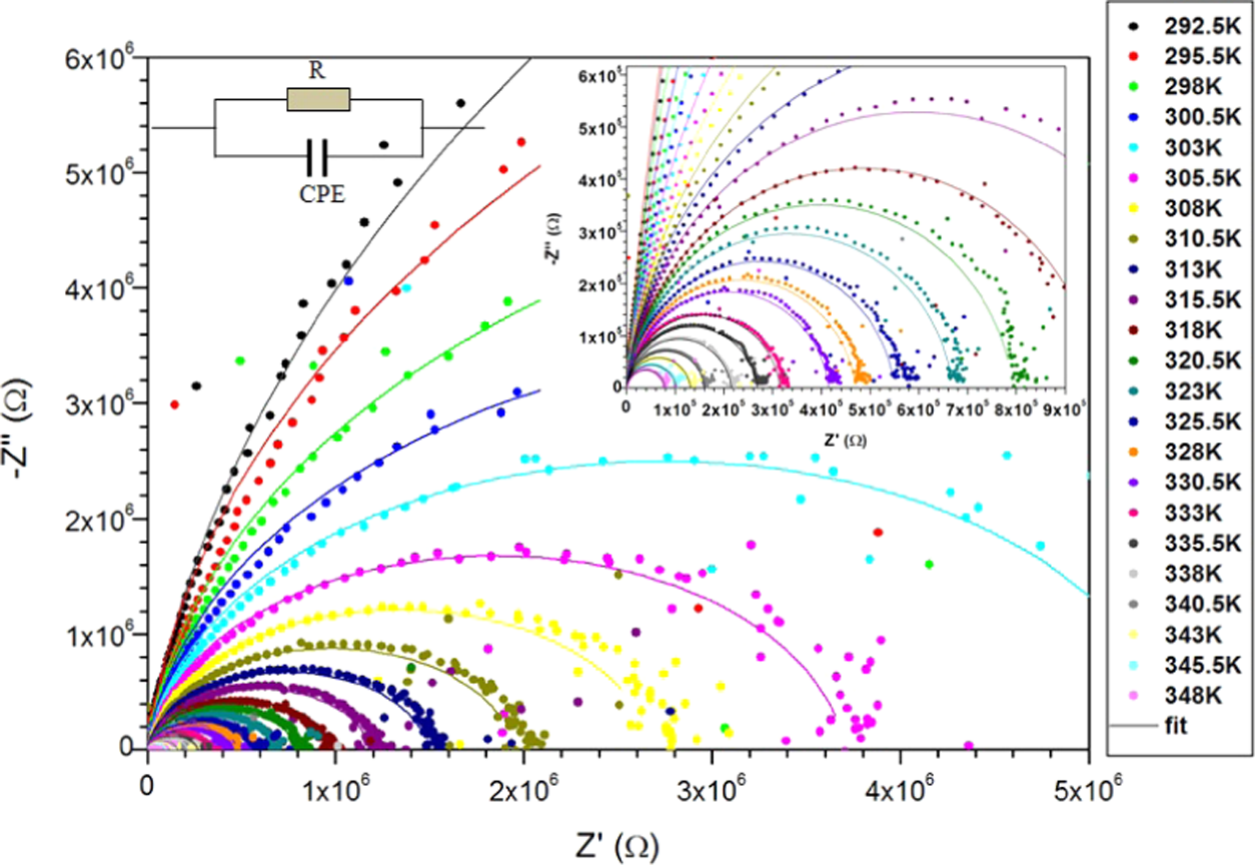
Nyquist plot for the impedance spectrum at different temperatures of PC film. Full lines are the fitting curves using the inserted equivalent electric circuit.

From Figure 15, we can see that when the frequency increases, the voltage signal becomes delayed relative to the current signal. At low frequencies, we observe resistance behavior (θ ≈ 0°). However, at high frequencies, we observe capacitance behavior (θ ≈ 90°). Therefore, the electrical equivalent circuit can be a parallel connection of a pure resistance (*R*) and an ideal capacitance (*C*), and thus, the relaxation follows the Debye model. However, in light of Figure 15, some discrepancies with the ideal Debye model can be seen. For example, at low frequencies (*f* → 0), phase angle θ ≠ 0, at high frequencies (*f* → ∞), phase angle θ ≠ −90°, and the Nyquist plot (Figure 16) shows semicircles with their centers under the real axis. Thus, the electric properties of our PC sample must be described by a modified Debye model^70−75^ in which the proposed equivalent circuit is a parallel set of a pure resistance (*R*) and a constant phase element (CPE), as shown in the inset of Figure 16. The CPE is defined as a frequency-dependent capacitance and has been introduced to improve the fit of the experimental impedance spectra by the equivalent electrical circuit models.^72,75^ CPE impedance is given by
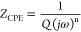
8where *Q* and *n* (0 ≤ *n* ≤ 1) are the parameters of the CPE. Case *n* = 1 represents an ideal capacitor, while case *n* = 0 stands for a pure resistor. The phase angle of CPE is θ = −*n* × 90°. The Nyquist plot of a resistor in parallel with a CPE is a semicircle depressed by an angle equal to  with respect to the *Z*′-axis. The real capacitance (*C*) is deduced from the universal capacitance (CPE) by the following formula^70,76^
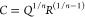
9We used a parallel set R-CPE equivalent circuit to fit the Nyquist plot for the impedance spectrum of our PC film (see Figure 16).

Figure 16 displays Nyquist diagrams (−*Z*″ versus *Z*′) of the PC sample at different temperatures. All of the experimental curves, for temperatures above 298 K, form a single semicircle, which shows the good dielectric property of the PC film. The semicircles have their centers below the real axis (skewed circular arcs), indicating a deviation from the ideal Debye-type behavior. This confirms the presence of a non-Debye relaxation phenomenon. This nonideal behavior can be attributed to several causes, such as different charge carrier types, different trap types, different conduction paths, etc.^72^ Further, the radii of these semicircles decrease with an increase of temperature, thereby confirming that PC has a negative temperature coefficient resistance behavior. This observation proves that the conduction processes are thermally activated and that PC has semiconductor behavior. At low temperatures below 298 K, we observe a linear response of *Z*″. It indicates the highly insulating behavior of PC at low temperatures. The theoretical fitting of the Nyquist diagrams is done by using the model circuit shown in the inset of Figure 16. The equivalent circuit parameters are listed in Table 5.

**Table 5:** Values of the Equivalent Circuit Parameters for Different PC Film Temperatures

temp° (K)	*R* (Ω)	*n*	*Q*(10^–12^Fs^n–1^)	*C* (pF)	φ (°)
292.5	(2.75 ± 1.00)10^7^	0.938 ± 0.007	5.72 ± 0.53	3.21	5.58
295.5	(1.76 ± 0.14)10^7^	0.951 ± 0.003	6.49 ± 0.26	4.07	4.41
298	(1.11 ± 0.28)10^7^	0.946 ± 0.003	6.29 ± 0.25	3.64	4.86
300.5	(7.79 ± 0.36)10^6^	0.942 ± 0.003	6.18 ± 0.26	3.35	5.22
303	(5.50 ± 0.42)10^6^	0.939 ± 0.003	6.08 ± 0.25	3.11	5.49
305.5	(3.71 ± 0.22)10^6^	0.937 ± 0.003	6.07 ± 0.25	2.96	5.67
308	(2.68 ± 0.07)10^6^	0.936 ± 0.004	6.10 ± 0.26	2.87	5.76
310.5	(1.95 ± 0.05)10^6^	0.934 ± 0.004	6.12 ± 0.27	2.75	5.94
313	(1.49 ± 0.03)10^6^	0.932 ± 0.004	6.12 ± 0.28	2.62	6.12
315.5	(1.19 ± 0.03)10^6^	0.926 ± 0.004	5.78 ± 0.28	2.24	6.66
318	(0.93 ± 0.02)10^6^	0.922 ± 0.004	5.60 ± 0.27	2.00	7.02
320.5	(0.79 ± 0.02)10^6^	0.922 ± 0.004	5.67 ± 0.28	2.00	7.02
323	(6.69 ± 0.14)10^5^	0.920 ± 0.004	5.65 ± 0.28	1.91	7.20
325.5	(5.50 ± 0.10)10^5^	0.918 ± 0.004	5.62 ± 0.28	1.81	7.38
328	(4.71 ± 0.08)10^5^	0.917 ± 0.004	5.63 ± 0.28	1.76	7.47
330.5	(4.15 ± 0.06)10^5^	0.922 ± 0.004	6.03 ± 0.28	2.03	7.02
333	(3.15 ± 0.04)10^5^	0.922 ± 0.004	6.23 ± 0.30	2.05	7.02
335.5	(2.68 ± 0.04)10^5^	0.921 ± 0.004	5.72 ± 0.29	1.81	7.11
338	(2.16 ± 0.03)10^5^	0.908 ± 0.005	5.59 ± 0.29	1.41	8.28
340.5	(1.61 ± 0.02)10^5^	0.919 ± 0.005	6.52 ± 0.34	1.94	7.29
343	(1.32 ± 0.02)10^5^	0.915 ± 0.005	6.50 ± 0.34	1.78	7.65
345.5	(1.02 ± 0.01)10^5^	0.915 ± 0.005	6.68 ± 0.33	1.79	7.65
348	(0.79 ± 0.01)10^5^	0.912 ± 0.005	6.90 ± 0.37	1.72	7.92

As shown in Figure 16, the Nyquist diagrams are well adjusted by the electrical equivalent circuit (R-CPE). Furthermore, the values of the equivalent circuit resistance (*R*) are very close to those obtained using the average (*Z*′_moy_) of the plateau’s *Z*′ values and the DC conductance, which proves that the electrical equivalent circuit (R-CPE) represents a suitable model for the PC film and perfectly fits the properties of propolis. The resistance (*R*) values decrease significantly by increasing temperature, which is another proof of the semiconductor behavior of the PC thin-film sample. Furthermore, the *n* parameter is very close to unity, so the CPE compound of the electrical equivalent model can be considered as a capacitor. The extracted capacitance (*C*) is almost constant (∼pF) and rather low for the used PC film temperatures (292–348 K).

**Figure 17:**
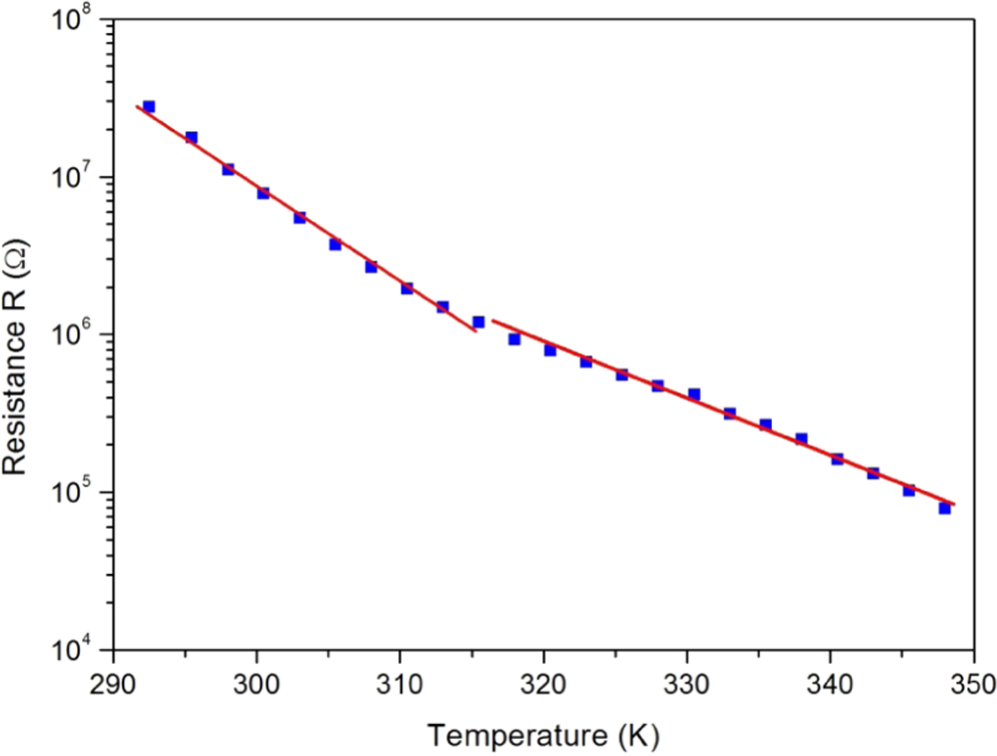
Variation with temperature of the resistance value (*R*) deduced from the equivalent electric circuit.

Figure 17 shows the plot of log *R* versus temperature. The curve is composed of two straight lines. This behavior of significant linear variation is suitable for a temperature-biocompatible sensor, and consequently, a PC film can be a potential bioactive layer in future medical thermometers.

## 4 Conclusions

The microstructural, optical, and electrical properties of the natural bioactive propolis complex (PC) films were studied. PC films were deposited by the drop-casting technique using an ethanolic solution of raw PC. Raw propolis was produced by *Apis mellifera* bee species on the island of Djerba in Tunisia. Thus, a stable, bioactive, green, and low-cost thin layer of PC was produced.

Morphological studies show that the PC thin films are smooth, dense, and continuous without pinholes or cracks and well cover the substrates surfaces. This confirms the effectiveness of the used growth technique despite its simplicity. The root-mean-square roughness value of the PC film is around 11 nm.

The PC film is opaque to UV radiation and the blue components of visible light. UV radiation is responsible for the oxidation of food and leads to nutrient losses, flavor degradation, and discoloration. Thus, PC films can be used in food packaging to prevent food deterioration. For green and yellow light (∼500–600 nm), PC films exhibit high absorption. However, for red and near-infrared radiation (∼600–2700 nm), PC films are transparent. In the frontier between near-infrared and mid-infrared wavelengths (∼2700–3200 nm), PC films reveal significant photosensitivity and so can be used as photosensors. The PC film’s energy gap was estimated as *E*_*g*_ ≈ 2.88 eV at room temperature, which, in principle, enables optoelectronic applications in the UV and blue ranges.

The electrical properties of the PC were investigated by complex impedance spectroscopy for a large frequency range (40 Hz to 40 MHz), at different temperatures (292–348 K), and in ambient air. The electrical study shows that the conductance of PC films increases with temperature and with frequency also, which is a sign of semiconductor behavior. At the low-frequency range, a large variation of the conductance (10^–8^–10^–5^ S) was observed for a relatively small variation of temperature (292–348 K). Therefore, the PC film exhibits potential applications as a safe and biocompatible negative-temperature-coefficient sensor and a thermal threshold controller in bioelectronics. The Nyquist plot displays a single skewed semicircle arc, which shows the good dielectric property of the PC layer and confirms the presence of a non-Debye relaxation phenomenon. We used a parallel combination of a resistor and a nonideal capacitor as an equivalent circuit model to fit the Nyquist plot for the impedance spectra of our PC film. Activation energies were deduced using several methods, and very similar values were obtained.

Therefore, in addition to the interesting applications of propolis in medical, pharmaceutical, and food industries due to its antibacterial, antiviral, antifungal, anti-inflammatory, anesthetic, antioxidant, antitumor (cytotoxic), immune-stimulating, wound-healing, and antiulcerogenic properties proved by other studies, propolis seems, in light of this study, to be a promising candidate for food packaging, optoelectronics, transparent electronics, biocompatible temperature sensing, and bioelectronics fields.

Finally, and in light of this study, we state that few published data exist on the physical properties of propolis. Therefore, more investigations are required both on the experimental and theoretical sides to shed more light on the amazing properties of this green bioactive material.
